# Circumferential penile defect reconstruction with pull-up double-opposing keystone-designed perforator island flaps: A case report

**DOI:** 10.1097/MD.0000000000018762

**Published:** 2020-01-17

**Authors:** Hyun Gun Lee, Soo Yeon Lim, Chi Sun Yoon, Kyu Nam Kim

**Affiliations:** a Department of Plastic and Reconstructive Surgery, Konyang University Hospital, University of Konyang College of Medicine, Myunggok Medical Research Center, Daejeon; b Department of Plastic and Reconstructive Surgery, Wonkwang University Hospital, Wonkwang University School of Medicine, Iksan, South Korea.

**Keywords:** keystone-designed perforator island flap, penile defect, reconstruction

## Abstract

**Rationale:**

The challenges with reconstruction of penile defects are plenty. In addition, no single and universally accepted reconstructive method exists for penile defect repair. Herein, we present the application of a circumferential penile shaft defect reconstruction using pull-up double-opposing keystone-designed perforator island flaps (KDPIFs) in one patient. This is the first case report of a circumferential penile shaft defect reconstruction using KDPIFs.

**Patient concerns:**

A 43-year-old man who injected petroleum jelly into his penis 10 years ago presented with multiple firm nodular mass-like lesions adherent to the overlying skin along the penile shaft. Our urologic surgeon removed the foreign bodies and performed a primary closure with undermining. However, wound dehiscence developed, and skin necrosis was exacerbated 5 days postoperatively.

**Diagnoses:**

We performed debridement, and the final post-debridement defect was circumferential (5.5 × 12 cm) from the base of the glans to the midpoint of the penile shaft.

**Interventions:**

We covered the defect using pull-up double-opposing KDPIFs (10 × 13 cm each) based on the hot spots of the superficial external pudendal artery perforators on each side from the suprapubic area to the scrotum.

**Outcomes:**

The flaps survived perfectly, with no postoperative complications. The patient was satisfied with the final outcome and had no erectile dysfunction or shortening of penile length after a 6-month follow-up.

**Lessons:**

We successfully reconstructed a circumferential penile defect with pull-up double-opposing KDPIFs both esthetically and functionally. Our technique can be a good alternative modality for extensive penile defect reconstruction.

## 1 Introduction

Penile defects are an uncommon and difficult problem to address physically and psychologically.^[1,2]^ Penile skin supplied from the external pudendal artery has characteristics such as thin skin, elasticity, pliability, and durability to withstand erection and friction. However, tissues having these properties are difficult to obtain.^[2]^ Thus, penile defect reconstruction is challenging. There are various reconstructive options including skin grafts, loco-regional flaps, and free flaps for the coverage of penile defects. Until recently, there has been no single and universally accepted reconstructive method for penile defect repairs. Reconstructive surgeons choose the proper method on a case-by-case basis because each technique has advantages and disadvantages. Furthermore, sometimes, the penile defect cannot be covered following previous reconstructive methods. Therefore, new or modified reconstructive techniques are warranted to cover the defect successfully and achieve better cosmetic and functional outcomes.^[3]^ The goals of penile reconstructive surgery are to provide reliable and durable coverage of the penis with minimal donor site morbidity both functionally and esthetically.^[2]^ Herein, we present and describe our modified keystone-designed perforator island flap (KDPIF) technique using pull-up double-opposing KDPIFs for the coverage of a circumferential penile shaft defect. To our knowledge, this report presents the first case of a reconstruction of a circumferential penile shaft defect using KDPIFs.

## 2 Case presentation

### 2.1 Case report

A 43-year-old man visited the Department of Urology at our hospital for the removal of foreign bodies in his penis. The patient readily proffered the information that he injected petroleum jelly subcutaneously into the penis with the intention of increasing its girth about 10 years ago. Physical examination revealed multiple firm nodular mass-like lesions adherent to the overlying skin along the penile shaft. Our urological surgeon performed a circumferential incision, removed the foreign bodies and fibrotic tissues, and performed a primary closure with undermining. However, wound dehiscence developed 5 days postoperatively and skin necrosis was exacerbated circumferentially. Thus, the patient was referred to the Department of Plastic and Reconstructive Surgery for wound management and reconstruction.

### 2.2 Surgical procedures

**Figure 1:**
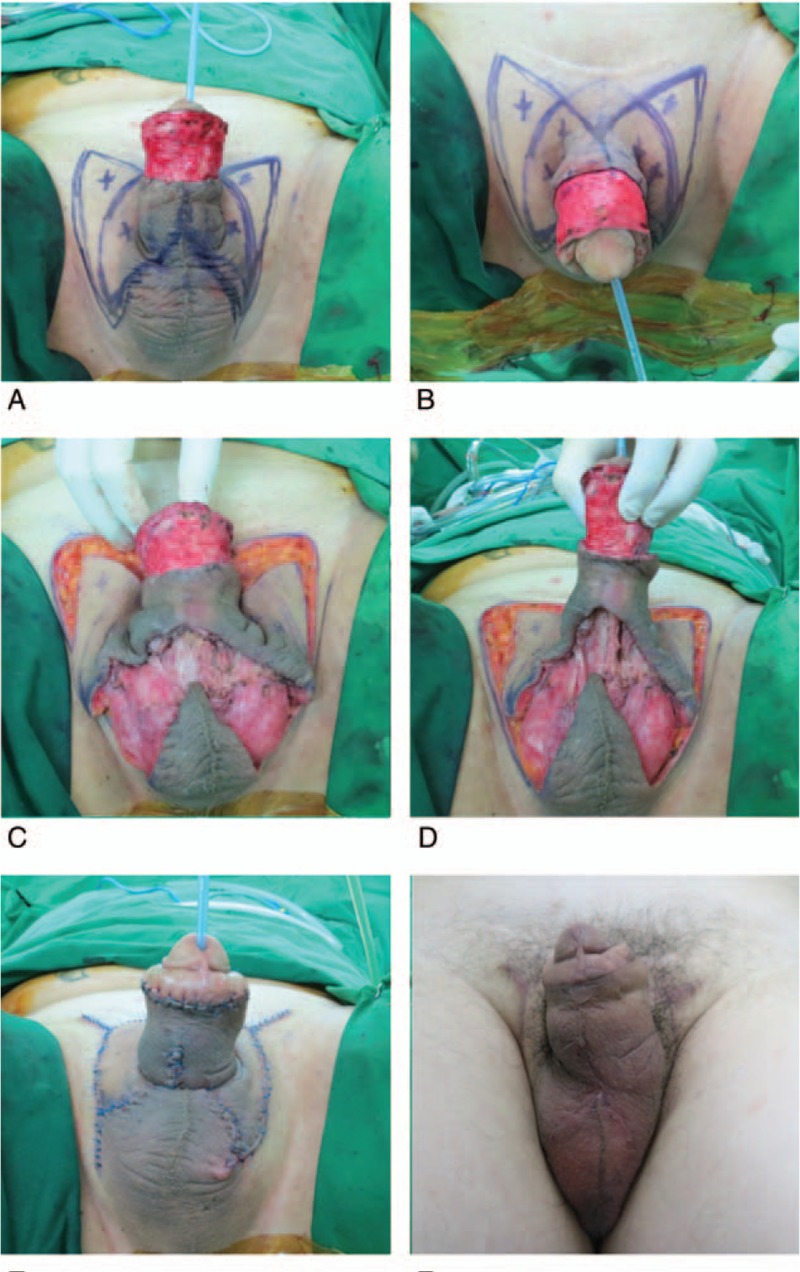
Clinical photographs. (A, B) The final post-debridement defect was circumferential (5.5 × 12 cm) and the double-opposing keystone-designed perforator island flap was designed for each side from the suprapubic area to the scrotum. (C, D) The flaps were elevated including the deep fascia with minimal undermining. The ring-shaped area of the flap surrounding the base of the penile shaft was freely undermined and pulled up to the defect. (E) Immediate postoperative image showed that the primary closure of the donor site, and tension-free in-setting of the flaps was achieved. (F) Six-month follow-up image showed maintenance of penile length and favorable scars.

We planned the debridement of necrotic tissues and flap coverage. The operation was performed with the patient in the lithotomy position under general anesthesia. We performed debridement using the Versajet II hydrosurgery system (Smith and Nephew, St. Petersburg, FL), and the final post-debridement defect was circumferential (5.5 × 12 cm) from the base of the glans to the midpoint of the penile shaft. We designed double-opposing KDPIFs (10 × 13 cm) based on the hot spots of the superficial external pudendal artery perforators on each side from the suprapubic area to the scrotum (Fig. 1A and 1B). The width of each flap was designed to be larger than the vertical width of the defect after considering the movement (pull-up) of the ring-shaped area of flaps surrounding the base of the penile shaft. Once the skin incision was made along the flap design, the dissection proceeded from the subcutaneous layer to the deep fascia (Buck's fascia of the penis and the external spermatic fascia of the scrotum). The fibrous subcutaneous septa and deep fascia were released using a monopolar device until the flap could be moved freely from the surrounding tissues. The margin of the flap was undermined minimally to preserve the integrity of the perforators. Then, the ring-shaped area of the flap surrounding the base of the penile shaft was freely undermined and pulled up to the defect (Fig. 1C and 1D). Primary closure of the donor site was achieved after tension-free in-setting of the flap (Fig. 1E). A mild compressive dressing was made with a foam material.

## 3 Results

**Figure 2:**
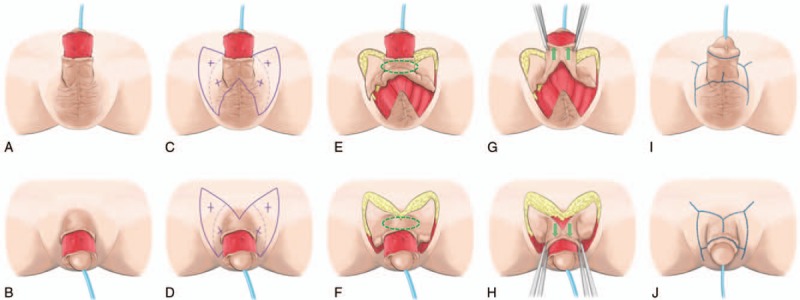
A stepwise schematic diagram of the pull-up double-opposing keystone-designed perforator island flaps (KDPIFs) for circumferential penile defect coverage. (A, B) Circumferential penile shaft defect. (C, D) Design of the double-opposing KDPIFs. (E, F) Elevation of the flaps and free undermining of the ring-shaped area (green-colored dotted oval line) of the flaps surrounding the base of the penile shaft. (G, H) Movement of the flaps (pulled up to the defect). (I, J) Tension-free in-setting of the flaps and primary closure of the donor site.

The flaps fully survived and there were no postoperative complications such as hematoma, seroma, infection, or dehiscence. The flap elevation time was 34 minutes and the operative time was 102 minutes. The patient was satisfied with the final outcome after a 6-month follow-up (Fig. 1F). Esthetically, there was good contouring and color matching without any shortening of the penis length or reduction in girth. Functionally, there was no scar contracture in both the penis and scrotum, and the patient had no difficulty in erectile and voiding function. Furthermore, he was able to achieve normal sexual intercourse. Figure 2 presents a stepwise schematic diagram of our operative procedures.

## 4 Discussion

We presented our experience of circumferential penile shaft defect reconstruction with pull-up double-opposing KDPIFs in one case, wherein good outcomes were obtained. As aforementioned, reconstruction of a penile defect is challenging, and various reconstructive methods have been developed. Skin grafts are commonly applied because they are a simple and popular option for small to large penile defects.^[2,4]^ However, skin grafts may often lead to contracture, stiffness, and unstable scars, which can cause erectile dysfunction or erectile pain.^[2,5,6]^ In particular, a skin graft is prone to result in suboptimal coverage for this size-changing organ.^[6]^ Furthermore, skin grafts often result in volume deficiency, which can cause an esthetically unacceptable appearance, and procuring graft tissue is a challenge for infected areas in contrast with a flap.^[7]^ Free tissue transfer, such as the radial forearm free flap, anterolateral thigh free flap, and superficial circumflex iliac artery perforator flap, has been used for expanded and total penile reconstruction.^[8,9]^ However, they may reflect overtreatment for partial or smaller penile defects and can be limited by the lack of skilled microsurgeons, the inability of centers to perform postoperative microsurgical monitoring and care, and the presence of comorbidities that prohibit lengthy operations.^[10]^ Moreover, the bulkiness of the flap, especially in obese patients, and the lack of sensation are potential limitations of free flap reconstruction.^[1,8,10]^

The scrotal flap is the most common local flap technique for penile defect coverage. Various types and modifications have been devised for extensive (generally circumferential) penile defects, which include the Cecil-Culp technique (penile scrotal implantation),^[11]^ advanced musculocutaneous scrotal flaps,^[3]^ bipedicled scrotal flaps,^[12]^ bipedicled anterior scrotal flap (Apron method),^[2]^ and bilateral scrotal flap with or without V-Y incision.^[1,5]^ The scrotal skin resembles the nature of the penile skin with regard to color and texture.^[2]^ Especially, the scrotal skin has high elasticity and pliability and is the most appropriate reconstructive option for penile shaft skin because its durability to withstand erection and friction can be acquired.^[2,3,5,12]^ Thus, reconstruction with scrotal skin flap in penile defects can provide both aesthetic and functional advantages over other reconstructive options, such as skin grafts, other local flaps, and free flaps. Among various types of scrotal flaps, some techniques, such as the Cecil-Culp technique and bipedicled scrotal flap, require a 2-stage operation.^[11,12]^ Other techniques, such as advancement musculocutaneous scrotal flap, bipedicled anterior scrotal flap, and bilateral scrotal flap, are performed as a one-stage operation.^[1–3,5]^ In case of the extensive and circumferential penile defect coverage, both dorsal and ventral areas of the defects are covered with scrotal skins in these scrotal flap techniques. However, the dorsal areas of penile defects are relatively far from the scrotum and, therefore, can have different skin properties from the scrotal skin. Thus, we consider that it is more suitable, with respect to reconstructive principles, to cover the dorsal penile defects with the skins that are much closer. In our case, we covered the circumferential penile shaft defect with the pull-up double-opposing KDPIFs. Each keystone flap was positioned on each side with respect to the midline from the suprapubic area to the scrotum as our design. Consequently, each dorsal and ventral defect in our case could be covered with the closest skin.

The KDPIF, devised by Behan in 2003, has been popularly used to cover cutaneous defects at various anatomical locations.^[10,13]^ KDPIFs have a curvilinear-shaped trapezoidal design and are essentially comprised of two end-to-side V-Y flaps. They are traditionally classified into four types: type I (skin incision only), type II (A, division of the deep fascia along the outer curvilinear line; B, division of the deep fascia and skin graft to the secondary defect), type III (opposing keystone flaps designed to create a double-keystone flap), and type IV (keystone flap with undermining of up to 50% of the flap subfascially).^[10,13]^ The KDPIF technique has the advantage of simple defect-adaptive design, easy reproducibility, safety, and short procedure time due to its inherent characteristics for minimal flap undermining and dissection.^[10]^ Several studies have described KDPIF reconstruction for perineal defects.^[14,15]^ The perineum is a perforator-rich area, and the scrotum receives plentiful blood supplies from the perineal branch of the internal pudendal artery, the external pudendal branches of the femoral artery, and the cremasteric branch of the inferior epigastric artery.^[1–3,7,12]^ In our case, we used double-opposing KDPIFs based on the hot spots of the superficial external pudendal artery perforators and performed much more undermining at the ring-shaped area of flap surrounding the base of the penile shaft in contrast with the minimal undermining concept of the original KDPIF technique. Nevertheless, all flaps fully survived with no complications. We considered that our surgical technique resulted in more reliable vascular perfusion due to the rich blood supply of the scrotum.

A previous study described that a V-Y incision on symphysis pubis in addition to the bilateral scrotal flap was effective for preventing shortening of penile length.^[5]^ In our surgical method, we consider that four end-to-side V-Y flaps of double-opposing KDPIFs had considerable effects on securing a sufficient penile length by advancements in both dorsal and ventral sides. Our patient had no tightening during penile erection and was satisfied with the postoperative penile length. Meanwhile, a previous study demonstrated that KDPIF reconstruction could guarantee favorable subjective and objective outcomes, which can be achieved by considering relaxed-skin tension lines, skin creases, and esthetic subunits in the design of the flap.^[10]^ Another study described that any single large local flap could be insufficient and unsatisfactory for extensive perineal defects that exceed the midline of the body, and multiple perforator flaps can achieve better functional and esthetic results.^[16]^ In the present case, we used 2 (double-opposing) keystone flaps in each side of the defect and designed the flaps with consideration for relaxed-skin tension lines, skin creases, and anatomical divisions. Thus, the final result showed a favorable outcome with the skin creases mimicking the natural creases.

Although we performed a successful penile defect reconstruction with KDPIFs, the current report has some limitations that should be acknowledged. We included only one case and used a non-randomized retrospective design. Thus, selection biases and the presence of confounding factors are unavoidable. Further prospective large-scale studies with other comparison groups are required to confirm the consistency of the observed favorable outcomes. Moreover, further studies are needed to address the limitations of the technique, including perfusion physiology and flap survival.

## 5 Conclusion

To the best of our knowledge, there has been no report of KDPIF reconstruction for isolated penile defects, and the current report presents the first case of a successful reconstruction of circumferential penile shaft defect using pull-up double-opposing KDPIFs both esthetically and functionally. Our technique can be a good reconstructive option to consider when covering extensive (generally circumferential) penile defects.

## Acknowledgments

We would like to thank Editage (www.editage.com) for English language editing and publication support.

## Author contributions

**Conceptualization:** Chi Sun Yoon, Kyu Nam Kim.

**Data curation:** Hyun Gun Lee.

**Formal analysis:** Hyun Gun Lee, Soo Yeon Lim.

**Investigation:** Chi Sun Yoon, Kyu Nam Kim.

**Methodology:** Chi Sun Yoon, Kyu Nam Kim.

**Writing – original draft:** Hyun Gun Lee.

**Writing – review & editing:** Soo Yeon Lim, Chi Sun Yoon, Kyu Nam Kim.
